# Electromyographic analysis of the traditional and spin throwing techniques for goalball games related to ball velocity for selected upper extremity muscles

**DOI:** 10.1186/s13102-024-00887-5

**Published:** 2024-05-09

**Authors:** Ayşenur Gökşen, Gonca İnce, Veysel Alcan

**Affiliations:** 1 Department of Physiotherapy and Rehabilitation, Faculty of Health Sciences, Tarsus University, Mersin, Türkiye; 2 Department of Coaching Education / Sport-Health Sciences, Faculty of Sports Sciences, Cukurova University, Adana, Türkiye; 3 Faculty of Engineering, Department of Electrical and Electronical Engineering, Tarsus University, Mersin, Türkiye

**Keywords:** Paralympics sport, Goalball, Surface electromyography, Performance analysis, Muscle activation

## Abstract

**Background:**

Goalball is a popular sport among visually impaired individuals, offering many physical and social benefits. Evaluating performance in Goalball, particularly understanding factors influencing ball velocity during throwing techniques, is essential for optimizing training programs and enhancing player performance. However, there is limited research on muscle activation patterns during Goalball throwing movements, needing further investigation to address this gap. Therefore, this study aims to examine muscle activity in sub-elite visually impaired Goalball players during different throwing techniques and visual conditions, focusing on its relationship with ball velocity.

**Methods:**

15 sub-elite Goalball players (2 female, 13 males; mean age of 20.46 ± 2.23 years) participated in the study. Muscle activity was evaluated with the Myo armband, while ball velocity was measured using two cameras and analyzed with MATLAB software. Different visual conditions were simulated using an eye band, and the effects of these conditions on muscle activation and ball velocity were examined.

**Results:**

The flexor muscles were found to be more active during the spin throw techniques with the eyes open (*p* = 0.011). The extensor muscles were found to be more active in the eyes-closed spin throw techniques compared to the eyes-open position (*p* = 0.031). Ball velocity was found related to the flexor muscles. Interestingly, no significant differences in ball velocity were observed between different throwing techniques or visual conditions (*p* > 0.05).

**Conclusions:**

Ball velocity, one of the performance indicators of the athlete, is primarily related to upper extremity flexor muscle strength rather than visual acuity. It has less visual acuity, but an athlete with more upper-extremity flexor muscle strength will have an advantage in Goalball game. The spin throw technique, which is reported to provide a biomechanical advantage for professional players in the literature, did not provide an advantage in terms of ball velocity for the sub elite players evaluated in our study. This knowledge can inform the development of targeted training programs aimed at improving technique and enhancing ball velocity in Goalball players.

## Introduction

Goalball is the most popular sport among visually impaired individuals, who highly prioritize engagement in physical activities [1, 2]. This team sport not only fosters social interaction but also enhances physical fitness and well-being [3]. Goalball is played with an eye band and a ball which has a buzzer and consists of two stages: defense and attack. During gameplay, all players, regardless of their visual acuity, wear eye band and rely on auditory cues to perceive the ball’s direction and proximity. In the defensive phase, players demonstrate agility by manoeuvring on the field to thwart the opposing team’s attempts to score. Upon gaining possession of the ball, a player transitions to the attaching phase, employing various throwing techniques. These techniques include straight throws without turning, as a bowling shot or rotational throws (360-degree arm rotation) resembling discus throws. In Goalball, ball velocity significantly influences the success rate of scoring attempts, with spinning motions (360-degree arm rotation) often preferred over traditional throws (without turning) for their ability to generate faster and more forceful shots [4]. The activation levels of certain muscles, such as the flexor and extensor muscles in the arm and hand, directly influence the speed and trajectory of the thrown ball [5, 6]. In spinning throws, for example, the coordination and synchronization of forearm muscles are essential for executing the rotational movement effectively. Higher activation levels in specific muscle groups, such as the flexor muscles, may contribute to generating greater torque and rotational force, resulting in increased ball velocity. Conversely, in traditional throws, where linear force is heavily applied, the activation patterns of forearm muscles may differ, with emphasis on stabilizing and propelling the ball in a straight trajectory. Therefore, understanding the activation patterns of forearm muscles is crucial for assessing ball velocity, particularly in different throwing techniques such as spinning and traditional throws in sports like Goalball. Surface EMG technology contributes to the assessment and improvement of athletic performance in sports science by providing a non-invasive and simple method for measuring forearm muscle activity which plays a significant role in generating the force required for ball propulsion during throwing movements muscle [7]. Identifying muscle activation patterns associated with best throwing performance can inform training strategies aimed at improving technique and enhancing ball velocity in sports-related movements. For instance, research in baseball pitching has utilized EMG technology to investigate the role of forearm muscle activation in pitch velocity and accuracy [8]. Physiological studies show that the muscles in the distal and proximal extremities work synergistically to create a movement pattern [9]. Although it is important to evaluate muscle activity during shooting to improve the athlete’s shooting performance and sports skills, it is also extremely important that the evaluation method is practical, accessible, and suitable for disability [10]. The integration of surface EMG with other technologies, such as Bluetooth low energy for real-time data transmission, further enhances its potential for remote monitoring, rehabilitation, and diagnostic techniques. In particular, new state-art wearable EMG devices offer a practical and non-invasive means of assessing muscle activation during dynamic tasks such as throwing movements, by enabling real-time measurement and recording of muscle activity [11]. Myo armbands, developed by Thalmic Labs as wearable and wireless EMG devices, offer dependable, robust, and low-cost EMG data, which allows humans to interact with computers. It has been used in various applications such as healthcare applications for people with disabilities with mobility problems, hearing-impaired people, multiple sclerosis, and children with motor impairment, upper-limb prostheses, controlling flying drones, robot movements or robotic prosthesis, gaming applications, virtual reality simulations, and human-computer interaction [12–15]. However, its use in determining performance in sports is limited, in particular, in the throwing performance of Goalball players [16]. To evaluate the relationship between forearm muscle (e.g., the flexors and extensors) activation and ball velocity in the Goalball, The Myo armband, with its capability to detect and interpret muscle activity, could potentially be used in sports performance analysis, including activities like handball or Goalball. It can provide useful information about muscle activation patterns and coordination during Goalball movements, and important factors in throwing performance [17].

Despite the growing interest in sports biomechanics and adaptive sports, there remains a notable gap in the literature concerning the relationship between forearm muscle activation and ball velocity in Goalball players. While Goalball serves as a prominent sport for visually impaired individuals, limited empirical evidence exists to elucidate the electrophysiological mechanisms underlying best throwing performance in this population. Specifically, the deficiency lies in the absence of comprehensive studies that systematically investigate how variations in forearm muscle activation patterns influence ball velocity during Goalball throwing movements. Understanding the nuanced interactions between muscle activation, throwing technique, and performance outcomes is paramount for developing targeted training interventions aimed at enhancing the effectiveness and efficiency of Goalball players’ throwing mechanics. Furthermore, the lack of empirical research in this area impedes the development of evidence-based training strategies tailored to the unique biomechanical and physiological needs of visually impaired players. By addressing this research deficit, the current study seeks to fill a critical knowledge gap and contribute valuable insights to the field of sports biomechanics and adaptive sports.

Therefore, in this study, we aim to evaluate the electrophysiological performance of forearm muscles in different visual conditions and Goalball penalty throwing techniques using the Myo armband. We hypothesize that flexor muscle group activity will predominate over extensor muscle group activity during shooting performance. Additionally, we hypothesize that spinning throws will confer an advantage over traditional throwing in terms of ball velocity and biomechanics. Lastly, we anticipate that the use of an eye band will adversely affect throwing performance.

## Methods

The present prospective, cross-sectional study followed with a strobe checklist and PICO–S (Population, intervention, comparison, outcomes, and study design) standard. The necessary ethics committee permission for the study was obtained from the Çukurova University Faculty of Medicine (date: 08.12.2023 decision no 139/66). All analyses of the study were evaluated in the performance analysis laboratory of the Çukurova University Faculty of Sports Sciences.

### Recruitment strategy and allocation of participants

**Fig. 1:**
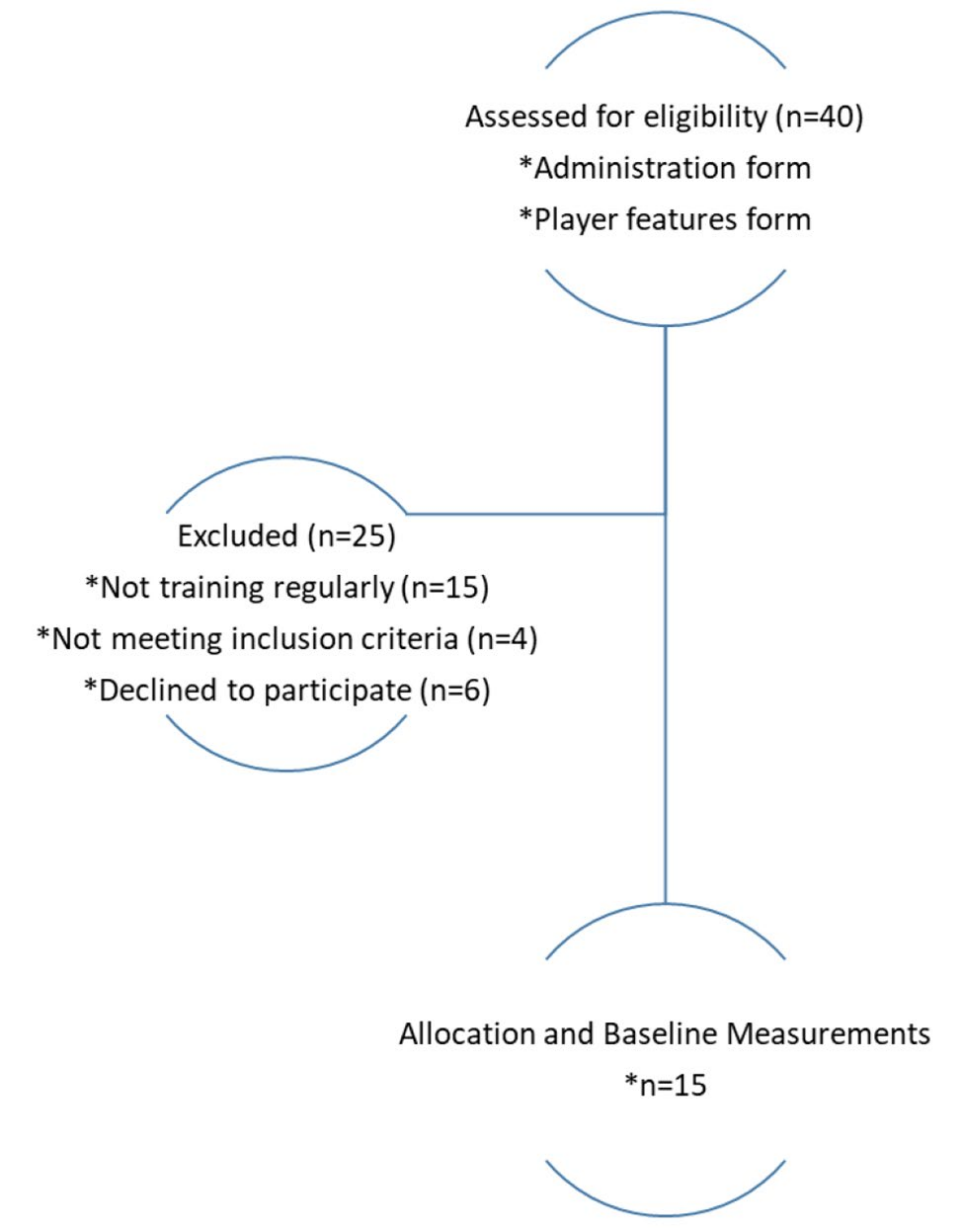
Flow chart

This study recruited visually impaired Goalball players aged between 18 and 25 who volunteered to participate. There are a total of forty Goalball players officially registered with the Ministry of Youth and Sports in Adana. These players were contacted and informed about the study, after which their suitability was assessed against the inclusion criteria. Fifteen players, who volunteered and met the inclusion criteria, were selected for participation. The stud comprised fifteen sub-elite Goalball players, consisting of thirteen men and two women, (Table 1). On average, the participants had been involved in Goalball for 5.4 ± 1.0 years and were engaged in regular training sessions. The mean age, height, and body mass index (BMI) of the participants were 20.46 ± 2.23 years, 174.14 ± 6.79 cm, and 24.05 ± 1.14 kg/m^2^, respectively. Visual impairment levels were classified according to visual acuity measurement. Visual acuity is the ability to see from a distance and distinguish details. Visual acuity is divided three groups as B1-B2-B3. B1 (Blind): People who have a visual acuity of 20/200 or less in the good eye after all corrections are called blind. B2-B3 (Low Vision) is defined that after all corrections, binocular vision is between 20/70 and 20/200. People who can only benefit from their vision in daily life with various assistive devices are called low vision. In a standard Goalball game, all disabled players, regardless of visual acuity level, play the game using an eye band. In this study, all vision levels were included. Sub-elite players with a minimum of three years of experience in Goalball were included in the study, while those who did not adhere to regular training were excluded from participation. In this study, “regular training” referred to the consistent participation of Goalball players in scheduled training sessions according to their respective training programs. Participants who had a musculoskeletal system injury related to the upper extremity that could affect throwing performance were excluded. In addition, participants with additional branches other than Goalball were excluded. The flow diagram is shown in Fig. 1.

### Data collection and synthesis

#### Evaluation of electromyographic muscle activity

**Fig. 2:**
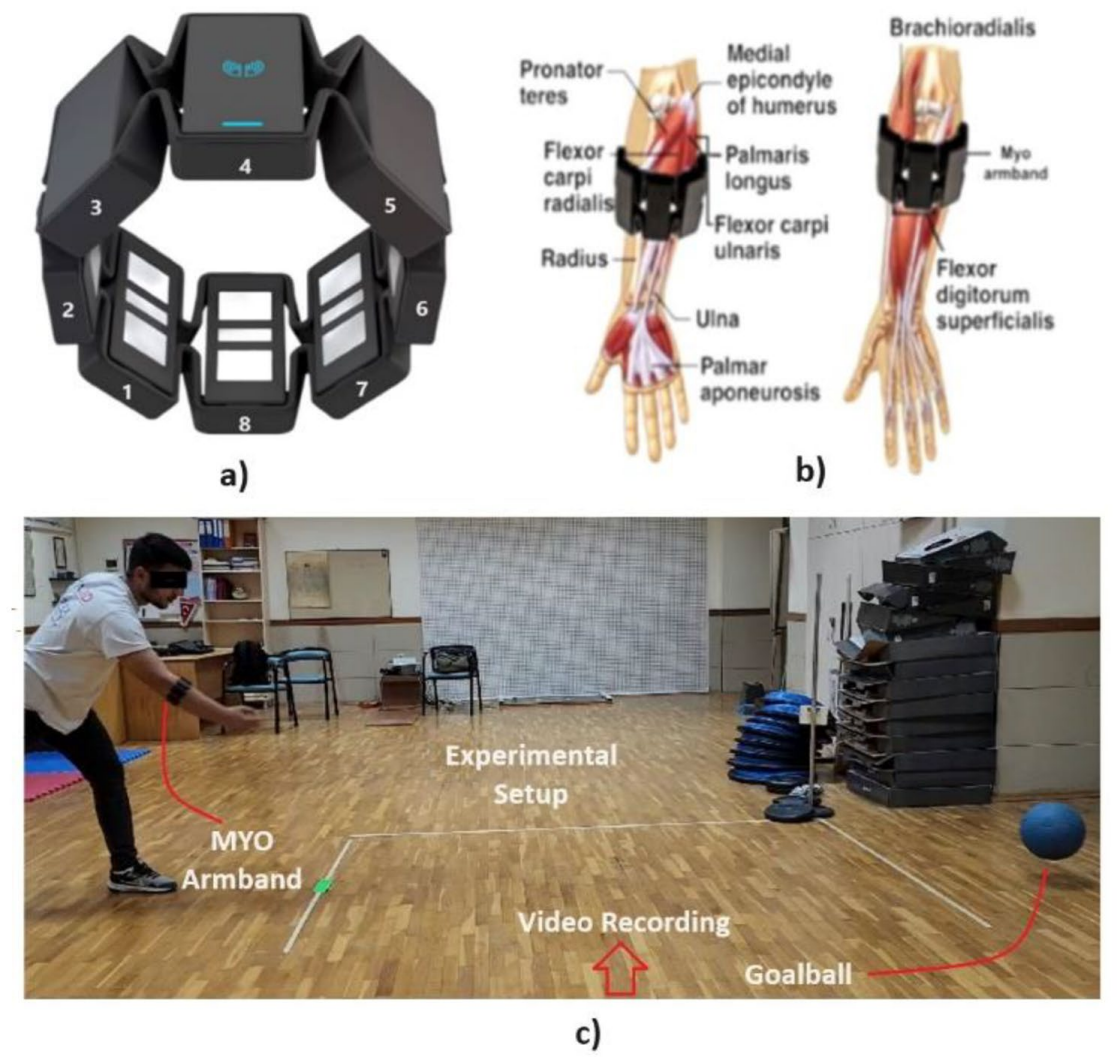
Illustration of the experimental setup and Myo Armband placement. (**a**) Myo Armband positioning on the forearm and below the elbow, showing sensor arrangement. (**b**) Specific muscles monitored by the Myo Armband during the study [18]. (**c**) Setup for quantitatively measuring ball velocity during Goalball throws, featuring a fixed 3-meter start-finish line on the ground

The Myo armband is provided with eight electromyographic electrodes, a 9-axes inertial measurement unit, and a transmission module. It acquires EMG data at 200 Hz. sampling frequency and sends the data via Bluetooth low energy technology, to other connected electronic devices such as laptops, personal computers, and mobile phones. In this study, the Myo Armband was securely fastened around the forearm and below the elbow. The adjustable straps were snugly wrapped, and each sensor (labelled 1 to 8) was strategically positioned on the armband. The central unit (number 4) features a sleek design with the emblem resembling a stylized leaf or symbol (see Fig. 2a) The Myo armband is designed to detect and interpret the electrical activity of muscles in the forearm. While it does not specifically measure individual muscles, it captures signals from a combination of muscles in the forearm region. The armband was placed on the upper forearm, as instructed by Thalmic Labs, approximately one inch distal from the tendon of the biceps brachii. The sensor 4 on an invisible line between the ring finger and index finger, and the extensor digitorum muscle. While sensors 2 to 5 are located in the posterior muscle group (the upper extensor muscles), sensors 6 to 8 and 1 overlap the anterior muscle group (the upper flexor muscles) [18]. The electrodes measure muscle activation of the brachioradialis, flexor digitorum superficialis, and the right extensor carpi radialis longus (See Fig. 2b). Electromyographic Analysis of forearm muscles during the ball-throwing were analyzed separately for two different conditions: eyes open and closed. Surface electromyography can distinguish between holding (force loading), throwing (active motion) and releasing Goalball (passive motion).

### Evaluation of ball velocity

Each participant was asked to throw the ball with straight and rotating throwing techniques, with randomly eyes open and closed conditions. To measure the speed of the ball quantitatively, a fixed start-finish line distance of 3 m was drawn on the ground (see Fig. 2c). Participants were made to throw the ball at the starting line. Those who did not follow the instructions properly had their throws cancelled and were asked to renew their throws after a 2-minute rest. The mobile phone camera was fixed in the middle of the start-finish line and video footage was taken. Then, each video was converted into frames with the program developed in MATLAB, and the time taken to reach the finish line from the first starting line was calculated (number of frames) [19].

A custom MATLAB interface was developed to facilitate data collection, real-time visualization, and offline signal processing. This interface dynamically updated these plots as new data was streamed from the Myo armband. The data included EMG signals from all 8 sensors was continuously recorded while participants throw the Goalball straight and spinning in eyes open and close conditions. Each movement was repeated two times to ensure an adequate dataset for subsequent analysis. All sEMG values were converted to an 8-bit unsigned integer with the scale factor in the device’s software. The range of potentials provided by the Myo armband is between − 128 and 128 in units of activation [20].

**Fig. 3:**
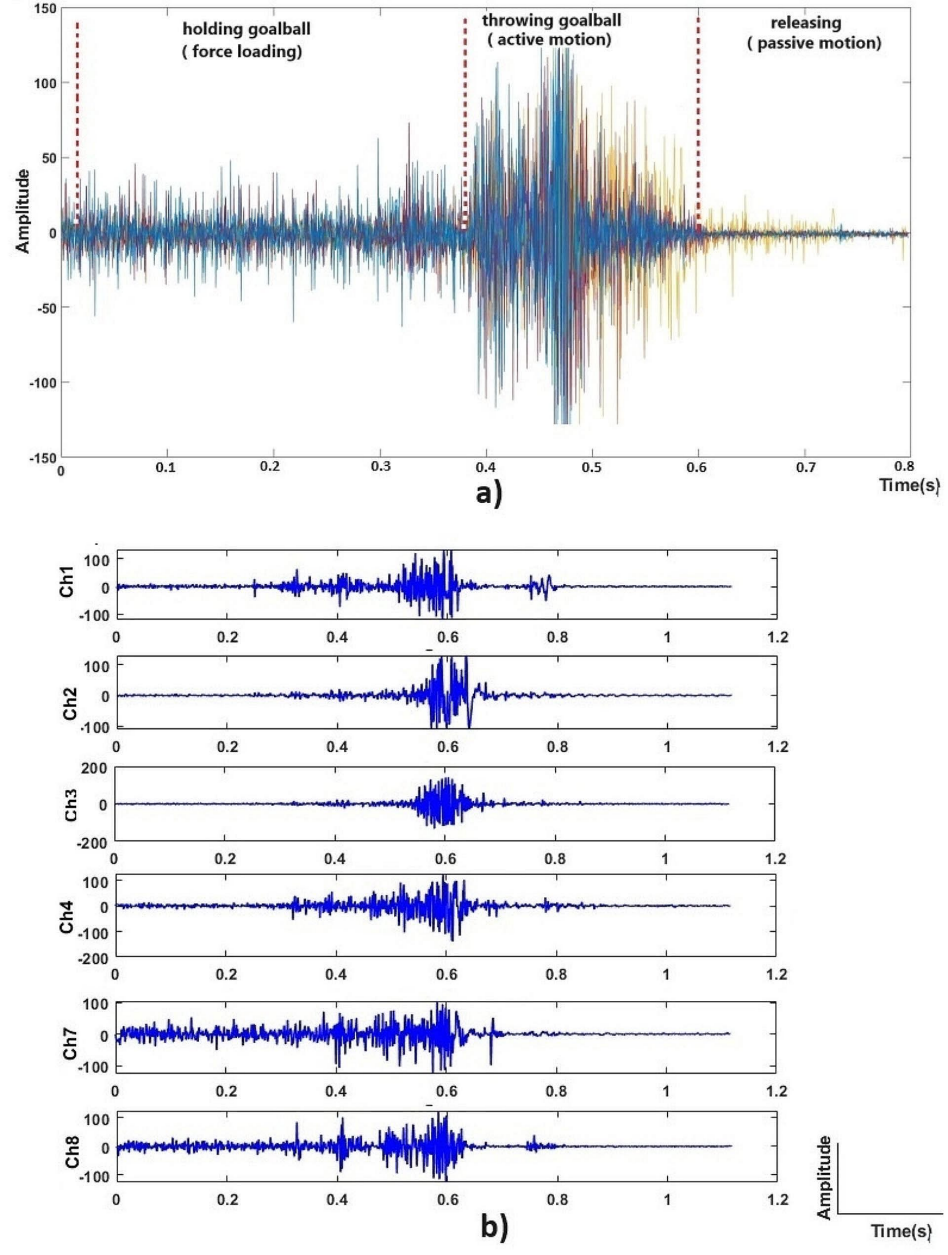
Muscle activations of forearm muscles recorded by Myo armband over 8 channels (sensors) (**a**) Display of all 8 channels together (**b**) sEMG signal recorded in each channel (sensor)

The signal processing was performed to identify muscle activation patterns and assess the effectiveness of throwing techniques. To remove noise and artifacts, a Butterworth bandpass filter with fourth orders was applied to focus on 20–500 Hz frequency range [21]. The data were then rectified to obtain the absolute value of the signal, emphasizing the magnitude of muscle activation by envelope (Fig. 3).

To quantify the amplitude of muscle activity during each movement, Root Mean Square (RMS) features were extracted from the pre-processed sEMG signals. For a given time window *T*, the RMS value *X*_RMS_​ for a channel *i* was calculated using the following equation:

$${X}_{{RMS}_{i}}=\sqrt{\frac{1}{T}} {\int }_{0}^{T}{x}_{i}{\left(t\right)}^{2 }dt$$

where *xi*​(*t*) is the rectified EMG signal for channel *i*.

This process was repeated for each channel of the Myo armband, resulting in a set of RMS features corresponding to different muscle groups.

### The protocol of Goalball players throwing the ball performance in the laboratory

In goalball, the act of throwing is such that offensive players move three steps forward, usually linearly (frontal throw) or rotationally (spin throw) and release the ball from the same sector of the 3-meter area [22]. Therefore, we used a distance of 3 m to throw the ball in the throwing protocol [23, 24].

### Statistical analysis

IBM SPSS Statistics 20.0 (IBM Corp. Released 2012. IBM SPSS Statistics for Windows, Version 20.0. Armonk, NY: IBM Corp.) was used for statistical analysis of the data. G*Power 3.1.9.4 software calculated the sample size. The predicted sample of 15 participants was adequate for a statistical power of %85. The Spearmen correlation coefficient (r) was calculated to determine the effect size of the significant results. The level of significance was determined as *p* < 0.05.

A kurtosis and Skewness Measure between ± 1.0 is considered excellent for most psychometric purposes. Since kurtosis and skewness value do not change between − 1.5 and + 1.5; The data did not comply with normal distribution [25, 26]. Physical and demographic characteristics were given as mean and standard deviation.

Comparison of the examined data and analysis in different situations using the Wilcoxon test; the relationship between the data was evaluated with the Spearman Correlation analysis test.

## Results

**Fig. 4:**
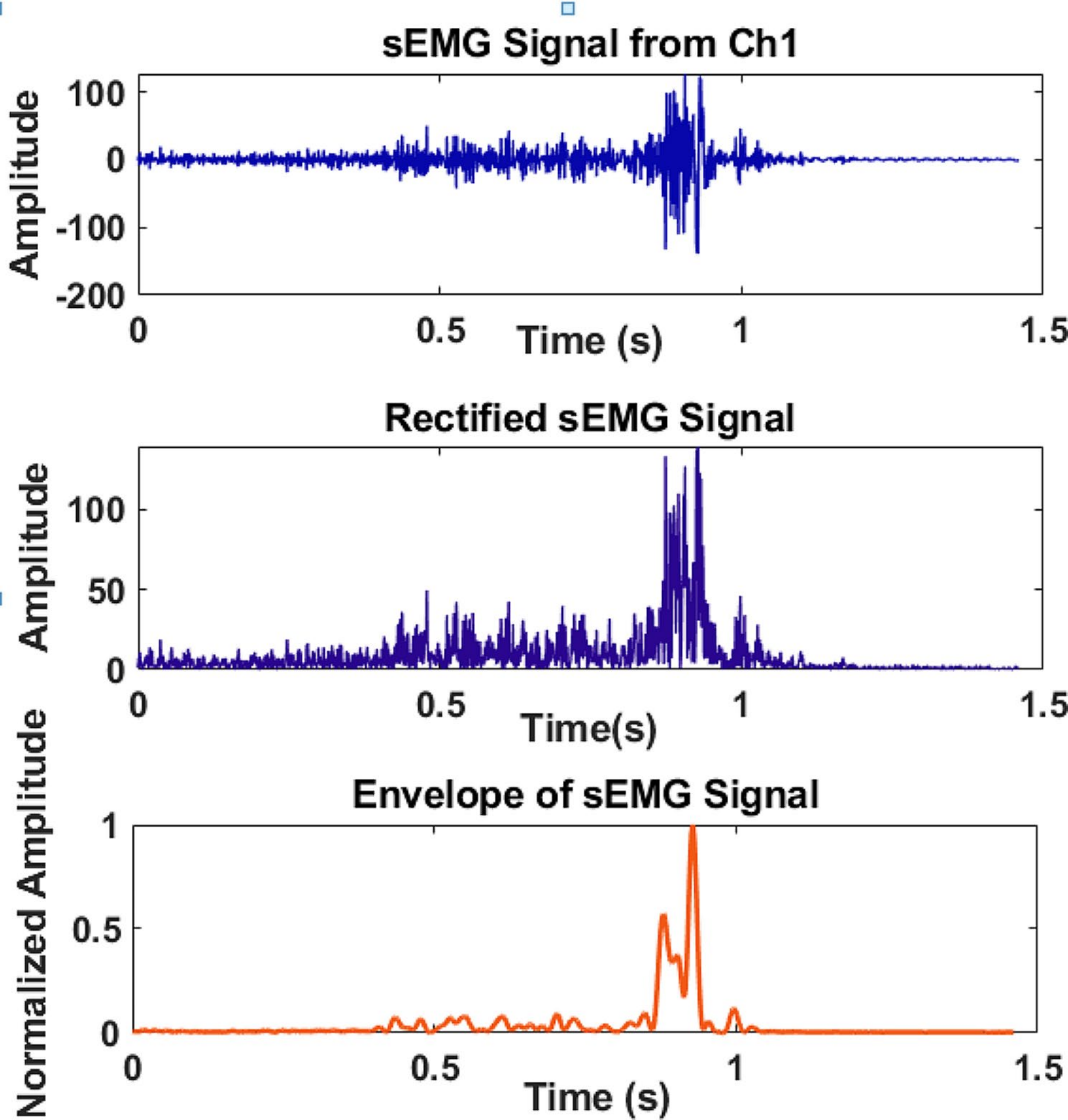
The signal pre-processing steps for each channel (sensor) recorded by Myo armband. It shows pre-processing steps of sEMG signal including filtered, rectified and envelope of the signals

The basic demographic and physical fitness characteristics of the players are presented in Table 1. Figure 4 shows how we observe muscle activity and patterns in selected muscles of the upper extremity in Goalball players in real-time. Figure 4a shows the three basic phases of activation of the arm muscles during straight throwing of the Goalball. A significant contraction was detected in the participant’s arm muscles while holding the ball and preparing to throw it (grip and weight of the ball). During the throwing of the ball, the muscles are fully active and contract very much. In the third phase, after throwing the ball, ridiculously small contractions are observed in the arm muscles while passive, that is, at rest. Figure 4b shows the activities of the muscle and/or muscle group recorded through each channel (sensor). The average ball-throwing speed of the players and muscle activation during ball-throwing are given in Tables 2 and 3. The extensor carpi radialis muscle was found more active in the closed eye position for the spin throw, (*p* = 0.031). Vision did not have any effect on muscle activations for the traditional throw, (*p* > 0.05). There was also no difference in muscle activation and ball velocity between spin and traditional techniques (*p* > 0.05), (Tables 2 and 3). Both traditional throws and spin throws were found associated with the flexor carpi radialis muscle (*p* = 0.028; 0.009), (Table 4).

**Table 1:** Demographic data of Goalball player

*n* = 15		X ± Sd
**Age (year)**		20.46–2.23
**BMI (kg/m**^**2**^)		24.05 ± 1.14
**Sports year**		5.4 ± 1.2
**Branch year**		5.4 ± 1.0
**Visual acuity**	**B1**	2
	**B2**	4
	**B3**	9
**Gender**	**Female**	2
	**Male**	13

n = number of Goalball player, BMI = body mass index, X = Mean, Sd = standard deviation

**Table 2:** Forearm muscle activity during Goalball throw and ball velocity

Muscles	Ball throw technique							
	Traditional Throw		*p* ^1^	Spin Throw		*P* ^2^	*P* ^3^	*P* ^4^
	OE	CE		OE	CE			
**Ch1 = Flexor carpi radialis**	0.028 ± 0.007	0.028 − 0.008	0.211	26.01 ± 100.65	0.029 − 0.008	0.460	0.334	0.638
**Ch2 = Extensor carpi radialis**	0.027 ± 0.009	330.56 − 0.008	0.211	0.027 ± 0.007	0.032 − 0.010	**0.031**	0.363	0.198
**Ch3 = Extensor digitorum communis**	0.027 ± 0.006	0.027 − 0.008	0.426	0.002 ± 0.006	0.029 − 0.009	0.140	0.649	0.510
**Ch4 = Extensor carpi ulnaris**	0.0025 ± 0.005	0.026 − 0.008	0.691	0.0246 ± 0.0048	0.025 − 0.004	0.495	0.363	0.397
**Ch7 = Flexor digitorum superficialis**	0.03 ± 0.007	0.0033–0.007	0.649	0.036 ± 0.01	0.034 − 0.007	0.307	0.099	0.754
**Ch8 = Palmaris longus**	0.029 ± 0.007	0.031 − 0.008	0.256	0.056 ± 2.05	0.030 − 0.007	0.256	**0.011**	0.600

Ch: Channel, OE: Open Eyes. CE: Closed Eyes. Ch: Channel. *p* < 0.05, Wilcoxon test. p^1^ = comparison visual condition for traditional throw. p^2^ = comparison visual condition for spin throw. p^3^ = comparison throwing technique for eyes open position. p^4^ = Comparison throwing technique for eyes closed position

**Table 3:** Comparison of the traditional and spin Goalball throw related to ball velocity

	Ball throw technique							
	Traditional Throw		*p* ^1^	Spin Throw		*P* ^2^	*P* ^3^	*P* ^4^
	OE	CE		OE	CE			
**Ball velocity (m/min)**	10.61 ± 4.42	29.83 ± 73.34	0.272	30.80 ± 79.65	34.28 ± 92.76	0.532	0.140	0.198

m: meter, min minute, *p* < 0.05, Wilcoxon test. p^1^ = comparison visual condition for traditional throw

p^2^ = Comparison visual condition for spin throw. p^3^ Comparison throwing technique for eyes open position

p^4^ Comparison throwing technique for eyes closed position

**Table 4:** Relation between ball velocity and muscle

Ball velocity/Muscle activation		Ch1 = Flexor carpi radialis	Ch2 = Extensor carpi radialis	Ch3 = Extensor digitorum communis	Ch4 = Extensor carpi ulnaris	Ch7 = Flexor digitorum superficialis	Ch8 = Palmaris longus
**Traditional throw**	*p*	**0.028**	0.407	0.378	0.610	0.316	0.465
	r	0.565	0.231	0.246	0.143	0.278	0.204
**Spin throw**	*p*	**0.009**	0.213	0.646	0.929	0.995	0.344
	r	0.647	0.342	-0.129	0.0250	-0.002	0.263

Ch: Channel. *p* < 0.05, Spearmen correlation test

## Discussion

In the present study, we aimed to bridge the gap in the literature by investigating the factors influencing ball velocity in Goalball players, particularly focusing on the activation patterns of forearm muscles during different throwing techniques. By utilizing wearable surface electromyography (sEMG) devices like the Myo armband, we have demonstrated the feasibility of assessing muscle activation in real-time during dynamic tasks, such as Goalball throwing movements and the factors affecting ball velocity in sub-elite Goalball players. According to our knowledge, this is the first study to evaluate throwing performance in Goalball players using Myo armband. Previous Studies in the literature have shown that the increase in ball velocity is directly proportional to the probability of throwing which results in a goal [27]. Therefore, in this study, we examined the factors affecting the ball velocity during the electrophysiological analysis of the throwing motion different throwing techniques, and different vision conditions.

### The relationships of muscle activation patterns and ball velocity

In this study, we found that the flexor muscle activity was related to ball speed. The first hypothesis of our study was confirmed with this result. This finding could be of importance for understanding the role of muscle activations and strength in the training of Goalball players. The results indicated that strength exercises targeting the upper extremity muscles may have a positive impact on improving throwing technique for visually impairment/blind Goalball players which is crucial for sports performance. Although muscle activation patterns during the throwing movement have been studied in different sports branches [28–30]. there are no studies on Goalball. Regarding Goalball players, previous studies commonly evaluated body composition, physical fitness, and aerobic capacity. Therefore, our result aligns with previous research highlighting the role of muscular activation in sports performance [31–33]. By discussing our findings about these studies, we can emphasize the relevance of our research within the broader context of sports science because it is particularly important to know muscle activation patterns to improve players’ training programs. Wearable EMG devices can help in the sports sector by providing muscle-usage indicators and live feedback to players, allowing them to identify areas for improvement and measure their progress. In the present study, muscle activation during the throwing movement was evaluated. The fact that flexor muscle activation was related to ball velocity can be explained by the fact that the agonist pattern during the throwing movement is the flexor pattern [4, 10, 32]. It was also found that the extensor muscles were significantly more active during the eye-closed spin throw. As it is known, spin throwing is a technique that requires more coordination and balance [34]. Antagonist muscle activity is higher during movements that require control. In order for the throwing movement to occur smoothly, the muscles must work in co-contraction [35]. The eccentric contraction of the antagonist muscle is extremely important for the control of the movement [36]. Considering that the agonist movement pattern during the throwing movement is a flexor, it is a reasonable result that extensor muscle activity increases in a throwing performance that requires more coordination. Based on these results, it is thought that adding eccentric exercises to the training programs of Goalball players will increase throwing performance. It was observed that there was a significant deficiency in the training programs of Goalball players and the literature [37]. One study reported that physical fitness levels affect the performance of Goalball players. In one study, it was reported that an 8-week core stabilization training program improved the performance of players [38]. Since there is no study in the literature examining upper extremity muscle activation patterns in Goalball, studies on the other branches were examined. During bowling which has a throwing technique similar to Goalball, there was less muscle activity in the wrist flexor muscles (46.53%) compared to the wrist extensor muscles [39]. In discus throwing, the forearm flexor muscle group exerted the highest power compared to other muscle groups [37, 40].

Another result was that the throwing types did not have superiority over each other in terms of ball velocity. However, it was a surprising result that the ball velocity was not higher in the spin throwing. It was a result that did not confirm the hypothesis we initially established. Studies in the literature have reported that spin throwing is more advantageous in terms of ball velocity [4, 34, 41]. These results may be related to the throwing preferences of the players. The athlete develops agility in the throw he/she uses frequently [42]. This result may also have been caused by the fact that the players in our study were amateurs and the spin throw required higher skill. Elite goalball players with a high technical performance display a successful and creative game. Such players tend to prefer and develop their own techniques according to their level of readiness, rather than using the standard goalball technique [43]. However, it is thought that sub-elite goalball players may be limited to standard goalball techniques used in training. This may be the reason the straight-throwing technique, which is easier to do among standard goalball techniques, is preferred more frequently in the sub-elite group. For this reason, it can be suggested that it would be appropriate for sub-elite athletes and their coaches to include the rotating throwing (spinning) technique in their technical training. We can emphasize that sub-elite Goalball players’ frequent use of this throwing technique in their training program may have a positive effect on the throwing performances of players.

### Visual sensory effect on motor performance

The rule of using eye band during the match prevents visual acuity from being an advantage. Whether visual acuity creates a biomechanical advantage is an unanswered question in the literature [4]. Furthermore, unlike other paralympic sports, the competition of individuals with different visual acuities in the same category with eye band in Goalball has been a controversial issue [10]. Therefore, we wanted to investigate the effect of visual manipulation. Our result revealed that visual manipulation did not affect ball-throwing velocity, but it effects on muscle activation patterns in visually impaired Goalball players. The present study was found that the ball velocity did not change with or without the eye band. In fact, it is an expected result that visual manipulation changes ball-throwing speed and muscle activation patterns in non-disabled players. However, the same cannot be said for disabled individuals. In disabled individuals, the motor adaptation process can occur by compensating for the lack of visual sensation with other sensory inputs. The effect of perceptual comprehension on motor skills in learning is quite high [44]. In those players, the motor adaptation process may occur by compensating for the lack of visual sensation with other sensory inputs. The effect of perceptual comprehension on motor skills in learning can be quite high [44]. Therefore, the players can gain motor-sensory adaption during their training to improve their motor performance. Over the past decades, sensorimotor research has revealed complex relationships between sensory input and motor output, shedding light on the bidirectional nature of these interactions [45–47].

### Practical implications

The practical implications of our findings are manifold. Firstly, our study provides valuable insights into the relationship between forearm muscle activation and ball velocity in Goalball, elucidating the biomechanical mechanisms underlying optimal throwing performance. This knowledge can inform the development of targeted training programs aimed at improving technique and enhancing ball velocity in Goalball players. Furthermore, our research highlights the potential utility of wearable sEMG devices like the Myo armband in sports performance analysis, particularly in assessing muscle activation patterns during Goalball throwing. By offering a practical and non-invasive means of evaluating muscle activity, these devices can facilitate objective performance monitoring and feedback for players and coaches. Additionally, our study underscores the importance of considering visual conditions, such as the use of eye bands, in Goalball performance evaluation. Contrary to our initial hypothesis, we found that the use of an eye band did not significantly impact ball velocity. This finding has implications for Goalball training and competition protocols, suggesting that visual acuity may not be a decisive factor in throwing performance.

In conclusion, our research contributes to the advancement of knowledge in sports biomechanics and performance analysis, particularly in the context of Goalball. By elucidating the role of forearm muscle activation in ball velocity and exploring the practical applications of wearable sEMG devices, our study offers valuable insights for researchers, coaches, and practitioners working in the field of adaptive sports. We believe that our findings pave the way for future studies aimed at optimizing training strategies and enhancing performance outcomes in Goalball and other visually impaired sports disciplines.

### Limitations and Future Works

In this study, there were several limitations. Firstly, the study population was limited in participant size. Despite the presence of approximately 100 visually impaired clubs and players registered in the Adana provincial representative office for the visually impaired, only a portion of these individuals actively participated in sports. This limited participation can be attributed to several factors such as educational commitments, familial responsibilities, and occupational engagements. Additionally, visually impaired players engage in a diverse range of sports activities beyond Goalball, including football, athletics, swimming, weightlifting, judo, and chess. While Goalball was chosen for its higher participation rate, the presence of players involved in multiple sports disciplines further constrained the sample size. Although efforts were made to address this limitation, the small sample size restricts the generalizability of the study findings. Another limitation pertains to the use of the Myo armband for athlete performance evaluation. As a wearable device, the Myo armband has a limited diameter, which restricts its ability to capture muscle activation data from broader areas of the body. While this limitation does not significantly impact angular evaluations, it does pose challenges in obtaining comprehensive muscle activation data, particularly from shoulder-related muscles. Consequently, the study’s findings may not fully capture the complete spectrum of muscle activation patterns during Goalball throwing movements. Furthermore, the literature lacks comprehensive insights into the training programs tailored specifically for Goalball players. Although this study offers valuable contributions by investigating the relationship between muscle activation and throwing performance in Goalball, future research endeavors should focus on evaluating the efficacy of targeted training interventions in enhancing Goalball players’ performance outcomes. Specifically, the incorporation of eccentric exercises into training regimens may hold promise for optimizing throwing performance by facilitating movements that demand greater control and precision.

In future works, larger-scale studies involving diverse populations of Goalball players, including elite players and those with varying levels of experience, could provide a more comprehensive understanding of the relationship between muscle activation and throwing performance. Additionally, longitudinal studies tracking players’ progress over time could elucidate the effects of training interventions on muscle activation patterns and performance outcomes. Furthermore, integrating advanced biomechanical analyses could provide deeper insights into the kinematics and kinetics of Goalball throwing techniques.

## Conclusions

In this study, we aimed to investigate the relationship between forearm muscle activation and ball velocity in visually impaired Goalball players. Our findings suggest that strength exercises targeting the upper extremities (concentric and eccentric) may have a positive impact on improving throwing technique and ball velocity in visually impaired goalball players, thereby enhancing their competitive performance. Contrary to our initial hypothesis, spin throwing did not significantly enhance ball velocity for the sub-elite players assessed in our study. It can be suggested that it would be appropriate for sub-elite athletes and their coaches to include the rotating throwing (spinning) technique in their technical training. We can emphasize that sub-elite Goalball players’ frequent use of this throwing technique in their training program may have a positive effect on the throwing performances of players. This study also highlights the potential utility of wearable surface electromyography (sEMG) devices, such as the Myo armband, in sports performance analysis. These devices offer a practical and non-invasive means of evaluating muscle activation patterns during dynamic tasks like Goalball throwing, enabling objective performance monitoring and feedback for players and coaches.

## Data Availability

The datasets used and/or analysed during the current study are available from the corresponding author on reasonable request.

## Abbreviations

sEMG: Surface electromyography
RMS: Root Mean Square
Hertz: Hz
